# Photobiomodulation Therapy for Neurosensory Disturbances in Orthognathic Surgery Patients: A Systematic Review

**DOI:** 10.3390/life15010111

**Published:** 2025-01-16

**Authors:** Aneta Olszewska, Mateusz Wolny, Julia Kensy, Agnieszka Kotela, Agata Czajka-Jakubowska, Jacek Matys

**Affiliations:** 1 Department of Orthodontics and Temporomandibular Disorders, Poznan University of Medical Sciences, 61-701 Poznan, Poland; anetaol@ump.edu.pl (A.O.); mz.wolny2@gmail.com (M.W.); a.czajka-jakubowska@ump.edu.pl (A.C.-J.); 2 Faculty of Dentistry, Wroclaw Medical University, 50-425 Wroclaw, Poland; julia.kensy@student.umw.edu.pl; 3 Medical Center of Innovation, Wroclaw Medical University, Krakowska 26, 50-425 Wroclaw, Poland; agnieszka.kotela@student.umw.edu.pl; 4 Department of Dental Surgery, Wroclaw Medical University, 50-425 Wroclaw, Poland

**Keywords:** LLLT, orthognathic surgery, paresthesia, PBM, physiotherapy

## Abstract

Background: Sensory disturbances and acquired paresthesia constitute a significant proportion of complications following orthognathic surgery. This systematic review examines the application of photobiomodulation (PBM) in managing these complications and its efficacy in promoting sensory recovery. Methods: In November 2024, a comprehensive digital search was performed across reputable databases, including PubMed, Web of Science, and Scopus, using carefully selected search terms: “orthognathic surgery” AND (physiotherapy OR physical therapy OR laser OR LLLT OR PBM OR light OR LED OR acupuncture) AND (nerve OR neurosensory OR paresthesia). The search adhered to the PRISMA guidelines. Of the 424 articles initially identified, 14 met the inclusion criteria and were included in the review. Results: The review focused on diode laser therapy for treating inferior alveolar nerve injuries, with most studies targeting this nerve and exploring diverse wavelengths, protocols, and surgical contexts, including orthognathic surgeries. Significant improvements were observed in tactile sensitivity, pain perception, and mechanical responsiveness. Factors such as earlier initiation of therapy, younger patient age, and higher treatment frequency were associated with improved outcomes. Conclusions: Low-level laser therapy emerges as a safe and effective intervention for enhancing neurosensory recovery following orthognathic surgery. However, the development of standardized treatment protocols and the conduct of larger-scale clinical trials are essential to optimize their clinical application.

## 1. Introduction

In recent years, there has been a growing focus among patients on enhancing facial esthetics and optimizing the functionality of the stomatognathic system [1]. Achieving these goals often requires more than orthodontic treatment alone, necessitating the integration of surgical interventions. Procedures such as orthognathic surgery have become increasingly common and accessible to a broader patient population [2]. These treatments are inherently complex, involving multiple stages, and the surgical procedures themselves are not without physiological impact [3]. Surgical interventions affect both soft tissues (e.g., mucous membranes, muscles, nerves, and blood vessels) and hard tissues (e.g., bones and teeth). Given the high innervation and vascularization of the operative area, there is an increased risk of complications, including pain, swelling, delayed wound healing, and neurosensory disturbances (NSDs) [4,5]. Postoperative complications, including dysaesthesia, hypoaesthesia, and anesthesia, are prevalent and often distressing for patients, with up to 87% experiencing subjective NSDs, some of which may be permanent [6,7,8].

**Figure 1:**
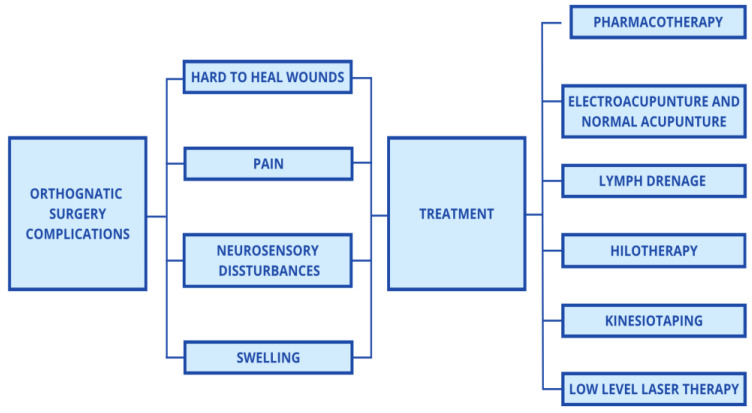
Complications after orthognathic surgeries and their possible treatment.

To enhance recovery following surgical procedures, the most widely utilized and standard approach remains pharmacotherapy, including anti-inflammatory drugs, antibiotics, and analgesics [9,10,11,12]. However, this conventional method is associated with several undesirable side effects, including gastrointestinal complications, hepatotoxicity, skin rashes, neutropenia, and postoperative nausea and vomiting [13,14,15]. As a result, alternative methods are gaining traction, such as kinesiotaping, hilotherapy, vitamin C supplementation, manual lymphatic drainage, and acupuncture (including electroacupuncture) [16,17,18,19,20,21]. Among these emerging interventions, physical laser biostimulation—specifically low-level laser therapy (LLLT)—has gained attention for its potential to support postoperative recovery (see Figure 1).

Low-level laser therapy (LLLT), also known as photobiomodulation (PBM), is increasingly used in physical therapy and medicine to stimulate cellular processes to achieve therapeutic benefits. This technique involves precise or diffuse irradiation of body surfaces to facilitate cellular biostimulation [22,23,24,25]. LLLT enhances bioenergetics, modulates cell membrane potential, and disrupts the synthesis of prostaglandins and tumor necrosis factor (TNF). It activates signaling molecules, stimulates gene expression, regulates reactive oxygen species (ROS) levels, adjusts intracellular redox states and pH, and promotes nitric oxide (NO) release, supporting cell growth and repair [26,27,28,29,30,31,32,33]. The therapeutic advantages of LLLT include anti-inflammatory and analgesic effects, improved bone regeneration, expedited wound healing, reduction in trismus and edema, enhanced microcirculation and vascularization, endorphin release, and nerve regeneration [34,35,36,37,38,39]. Beyond orthognathic surgery, LLLT is effective in treating arthritis, sciatica, neuralgia, acne vulgaris, and periodontal diseases [28,40,41,42].

Photobiomodulation (PBM) has been used for decades to manage neurosensory disturbances (NSDs) following orthognathic surgeries, with continuous advancements in therapeutic techniques and patient recovery. While several systematic reviews have examined the effects of low-level light therapy (LLLT) on pain, trismus, edema, and NSDs in maxillofacial surgery [5,43,44,45], recent reviews specifically focusing on NSD recovery are lacking. This highlights the need to re-evaluate the role of LLLT in improving NSD outcomes and to consolidate current findings, aiming to standardize treatment protocols and enhance patient recovery.

## 2. Materials and Methods

### 2.1. Focused Question

This systematic review adhered to the PICO framework to formulate the research question [46]:

PICO Question: In patients undergoing orthognathic surgeries (Population), does laser therapy (Intervention) enhance neurosensory recovery (Outcome) compared to the natural postoperative recovery process (Comparison)?

### 2.2. Protocol

**Figure 2:**
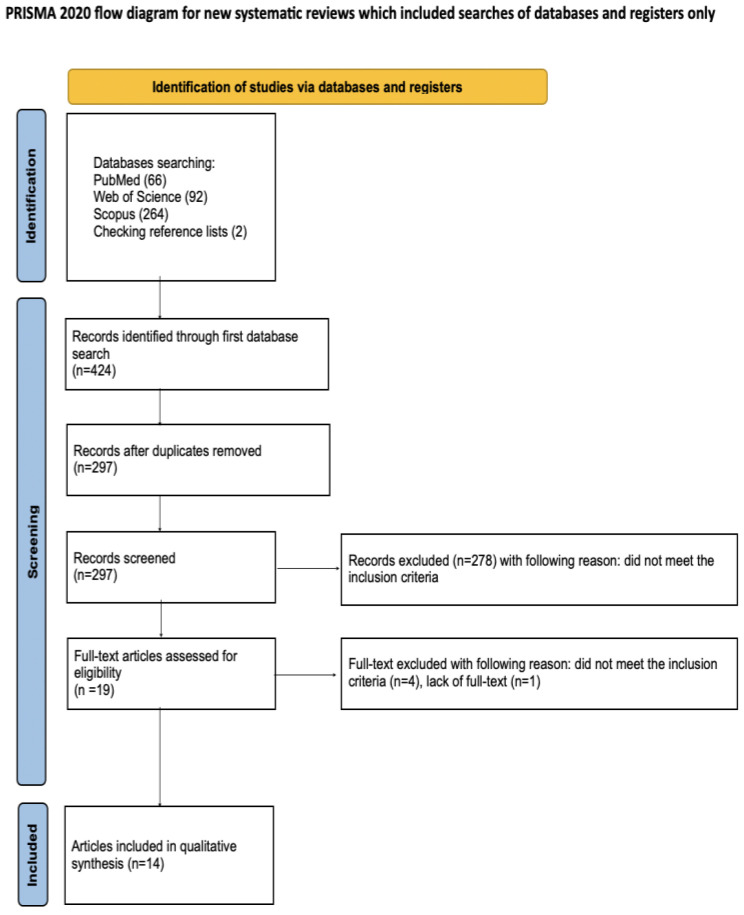
The PRISMA 2020 flow diagram.

The selection process for articles included in the systematic review was meticulously designed and documented in accordance with the PRISMA (Preferred Reporting Items for Systematic Reviews and Meta-Analyses) guidelines [47], as illustrated in Figure 2. The systematic review was registered on the Open Science Framework under the following link: https://doi.org/10.17605/OSF.IO/6XJ7T (accessed 16 December 2024).

### 2.3. Eligibility Criteria

Studies were deemed eligible for inclusion in the review if they met the following criteria [47]:

- Orthognathic surgery procedures;
- The use of photobiomodulation, low-level laser therapy, or LED light;
- In vivo studies;
- Studies with a control group;
- Case reports;
- Studies published in English;
- Prospective case series;
- Non-randomized controlled clinical trials (NRS); and
- Randomized controlled clinical trials (RCTs).

The reviewers established the following exclusion criteria [47]:

- Oral surgery procedures that were not orthognathic surgeries;
- Absence of laser treatment;
- Non-English papers;
- Opinions;
- Editorial articles;
- Review articles;
- No full-text access; and
- Duplicated publications.

No restrictions were applied regarding the year of publication.

### 2.4. Information Sources, Search Strategy, and Study Selection

In November 2024, a comprehensive search was performed in the PubMed, Scopus, and Web of Science (WoS) databases to identify articles that met the predefined inclusion criteria. The search targeted titles and abstracts, employing specific keywords to locate studies on laser therapy for neurosensory recovery following orthognathic surgeries. The search terms included: orthognathic surgery AND (physiotherapy OR physical therapy OR laser OR LLLT OR PBM OR light OR LED OR acupuncture) AND (nerve OR neurosensory OR paresthesia). Additionally, the reference lists of included articles were reviewed to identify further relevant studies. All searches adhered strictly to the eligibility criteria, and only studies with full-text availability were considered for inclusion.

### 2.5. Data Collection Process and Data Items

The articles that met the inclusion criteria were independently reviewed and extracted by three researchers (M.W., A.K., J.K.). The extracted data included the first author, year of publication, study design, article title, type of laser used in the study, and its effectiveness and outcomes related to the healing process and pain relief. The collected information was systematically organized and recorded in a standardized Excel spreadsheet.

### 2.6. Risk of Bias and Quality Assessment

In the initial phase of study selection, each reviewer independently screened the titles and abstracts to minimize potential bias. Cohen’s kappa (κ) test was employed to assess the level of inter-reviewer agreement [48]. Any disagreements regarding the inclusion or exclusion of articles were resolved through thorough discussions among the authors until a consensus was reached.

### 2.7. Quality Assessment

Three reviewers (M.W., A.K., J.K.) independently evaluated the quality of the included studies. The assessment considered key aspects such as study design, execution, and analysis, using specific criteria: a minimum sample size of 10 patients, the presence of a control group, randomization, blinding, and sample size calculation. Studies were scored on a 0 to 6-point scale, with higher scores indicating better quality. The risk of bias was categorized as follows: 0–2 points signified a high risk of bias, 3–4 points indicated a moderate risk, and 5–6 points represented a low risk. Disagreements in scoring were resolved through discussion among the reviewers until consensus was reached [47].

## 3. Results

### 3.1. Study Selection

The initial database search across PubMed (66 articles), WoS (92 articles), Scopus (264 articles), and reference lists (2 articles) yielded a total of 424 potentially relevant articles for the review. After the removal of duplicates, 297 articles remained for screening. During the initial screening of titles and abstracts, 278 articles were excluded for not meeting the inclusion criteria. Subsequently, 19 articles underwent a detailed full-text analysis. Of these, four articles were excluded for failing to meet the inclusion criteria, and one article was excluded due to the lack of accessible full text. Ultimately, 14 articles were included in the qualitative synthesis of this review [49,50,51,52,53,54,55,56,57,58,59,60,61,62].

### 3.2. General Characteristics of the Included Studies

This systematic review included an analysis of 14 studies encompassing various study designs. These comprised randomized controlled trials (RCTs), one retrospective analysis [56] involving 125 participants, one case report [57], and one prospective observational study [58] with six patients, all of whom received identical treatment protocols. Control groups were employed in 11 studies, utilizing either a split-mouth design [49,53,59,62] or separate control groups [50,51,52,54,55,60,61]. Sample sizes varied widely, ranging from a single case report [57] to larger cohorts of up to 40 participants [52].

Regarding surgical interventions, bilateral sagittal split osteotomy (BSSO) was the most commonly performed procedure, featured in seven studies [52,53,54,55,57,58,59,62], while sagittal split osteotomy (SSO) was reported in three studies [52,60,61]. More complex surgical approaches included combinations such as orthognathic surgery with genioplasty [57], BSSO with mentoplasty and Le Fort I osteotomy [50], and BSSO with genioplasty and Le Fort I osteotomy [51]. Additionally, one retrospective study encompassed a broader range of procedures, including orthognathic surgery, third molar extractions, dental implant placement, trauma-related surgeries, and other minor oral surgical procedures [56].

In terms of laser procedures, most studies utilized a single diode laser wavelength approach, with ten studies employing a specific wavelength consistently throughout their treatment protocols [49,50,51,52,53,55,57,58,59,61]. However, some researchers adopted more complex approaches. Baydan et al. implemented two different wavelengths (904 nm and 650 nm vs. 940 nm) in separate treatment groups [54]. De Oliveira et al. employed a two-stage protocol, starting with an 808 nm laser and transitioning to a 660 nm wavelength if necessary after ten sessions [56]. One study used a dual approach, applying a 660 nm laser intraorally and an 810 nm laser extraorally [62]. Additionally, Mohajerani et al. combined laser therapy with LED treatment [60].

The wavelengths used across the studies ranged from 780 nm to 940 nm, with the 808–830 nm range being the most common. Two studies utilized a wavelength of 780 nm [49,59]. The 808 nm wavelength was used in two studies [53,57], while 810 nm was implemented in another two [52,55]. Single studies explored various other wavelengths: 820 nm [61], 820–830 nm [58], 830 nm [50], and 940 nm [51].

In connection with the affected nerve areas, ten studies specifically focused on the inferior alveolar nerve [49,52,53,54,55,56,58,60,61,62]. Some studies examined broader regions: Travassos Prazeres et al. [50] investigated both the upper and lower lip, along with the chin area, while Pimenta D’Avilla et al. [51] focused on an extensive region extending from the preauricular area to the mandibular body, including the paranasal areas, lips, and chin. Morais Filho et al. [57] targeted the nose, nasal folds, lower lip, and chin regions, whereas Santos et al. [59] concentrated on the mandibular region (see Table 1).

### 3.3. Main Study Outcomes

The efficacy analysis predominantly demonstrated positive outcomes in laser-treated groups, with the majority of studies reporting significantly superior results compared to control interventions. Notably, only one study found no statistically significant differences between the intervention groups [49].

Assessment methods included objective measures alone [50,62], subjective measures alone [56], or combined approaches [49,51,52,53,54,55,57,58,59,60,61]. Evidence suggests recovery rates of up to 85% in laser-treated groups compared to 70–75% in control groups [55]. Multiple studies reported significant improvements in two-point discrimination [52,58,62], tactile sensitivity [53,59], pain response [51,55], and mechanical sensitivity [55,61]. Recovery patterns varied by anatomical location, with the lips and teeth demonstrating better recovery outcomes compared to the chin region [53]. Complete resolution of paresthesia was observed in a laser-treated group within six months post-surgery [54]. Follow-up periods ranged from immediate postoperative assessments to two years [55].

Several factors were identified as influencing treatment outcomes. Early intervention, particularly within 30 days post-surgery, was associated with improved recovery outcomes. Patient age also played a significant role, with younger individuals (14–25 years) exhibiting superior recovery rates. An analysis of treatment frequency indicated that weekly sessions were more effective than bi-weekly treatments [56]. Furthermore, in studies employing a split-mouth design, laser-treated sites demonstrated faster recovery compared to untreated sites [53,59,62] (see Table 2).

### 3.4. Quality Assessment of the Included Studies

Ten articles included in the review were predominantly of high quality, with scores of 5/6 [53,54,55,61] or 6/6 [49,51,52,59,60,62]. However, three studies [50,57,58] were categorized as low quality. Additionally, only one study, scoring 4/6 points, was identified as having a moderate risk of bias [56] (see Table 3).

**Table 2:** Detailed characteristics of included studies.

Authors	Type of the Surgery	Affected Area/Nerve	Method of Assessing Sensory Disorders	Laser Type	Laser Parameters	Results (Restoration of Sensation in Areas Affected by Paresthesia)
de Oliveira [49]	Combined orthognathic surgery and genioplasty	Inferior alveolar nerve	- Mechanical evaluation with the brush #2 and #12, - 2-point discrimination test calibrated on 5 mm and 10 mm; - Electric pulp test on lower 2nd molars, 1st premolars, and central incisors.	Diode laser LaserHand, (MMOptics, São Carlos, SP, Brazil)	780 nm, in contact mode, 70 mW, with a spot size of 0.04 cm^2^, 6 s/point, 0.42 J/point, and 10 J/cm^2^, 2× per week	No differences in responses between laser and the control group.
Travassos Prazeres [50]	Bilateral sagittal split osteotomy (BSSO), mentoplasty, Le Fort I	Upper lips in Le Fort I patients Lower lips and chin in others	- Superficial mechanical sensitivity - surface swab; - Deep mechanical sensitivity—clinical clamp; - Thermal sensitivity—ice cube; - Paresthesia evaluation—rated from 1 intensive to 4 absent through the patient’s response to the stimuli.	Diode laser GaAlAs	830 nm, 50 mW, 0.6 spot, 20 J/cm^2^ per session	At the 12th session, test group showed lower paresthesia during deep mechanical and thermal sensitivity than control group and faster return of the studied sensitivities. The chin region had higher paresthesia and slower regression than the lower lip. The deep mechanical sensitivity decreased first compared to the superficial and thermal.
D’avilla [51]	Le Fort I Osteotomy, Bilateral sagittal split osteotomy (BSSO), genioplasty	From the preauricular to the mandibular body region, paranasal region, upper and lower lip, chin, Alveolar inferior nerve.	- Contact detection with a fine paintbrush; - Visual analog scale (VAS).	Diode laser InGaAsP semiconductor (Epic X, Biolase)	940 nm diode laser with 50–60 Hz circular beam shape with continuous wave, 4000 mW, 7.1 cm^2^ area spot, 0.56 W/cm^2^, 21.12 J/cm^2^, 150 J total, 30 mm from the skin, for 5 s intervals, 37.5 s total per point (8 points total)	From 24 h up to week 3 laser group reported less pain than the control group; From baseline to week 4 the laser group displayed higher positive responses to the contact test, but without significant difference between experimental periods.
Esmaeelinejad [52]	Sagittal split ramus osteotomy (SSRO)	Inferior alveolar nerve	- Two point discrimination test—with calibrated drawing compass; - Thermal test—the heat or cool probe with water perception; - Pinprick test—sharp needle identification; - Patient’s satisfaction.	Diode laser	810 nm, 70 mW, 0.8 cm diameter spot size, 140 mW/cm^2^, 8.4 J/cm^2^, 60 s for each point, for 8 min and 67.2 J total irradiation	Control and test groups were able to detect heat and cool; Test group successfully identified the touch with a sharp needle after one year, showed better distinction of two separate sharp points, positive contact direction test and higher satisfaction.
Buysee Temrano [53]	Bilateral sagittal split osteotomy (BSSO)	Inferior alveolar nerve (25 points on average per side)	- The mechanoreceptor tests—brushing tested and control side: skin of the posterior and middle mandible area, inferior lip, labial commissure, chin, labial mucosa and vestibular gum; - The nociceptors tests—thermal tests with hot gutta-percha and endo-frost applied to the crowns of incisor, premolar, and molar; - Visual analog scale (VAS).	Low intensity infrared GaAlAs laser (A W. Laser II DMC—São Carlos—SP/Brazil)	808 nm laser, 100 mW, 0.0028 cm^2^ tip spot, 2 mm distance from the irradiated area, 0.028 cm^2^ area, 3.6 W/cm^2^, 2.8 J per point, 100 J/cm^2^, 28 s each point with the distance of 1 cm between points	No significant sensibility difference based on the type of stimuli; Improvement, higher perception and faster recovery from sensory disorders in the test group.
Baydan [54]	Bilateral sagittal split osteotomy (BSSO)	The inferior alveolar nerve—the part it supplies such as lower lip and chin area	Tests were performed at 6 timepoints (T0–T6). Area between lower lip and chin was divided into 9 fields for each side: - Brush test (yes/no); - Two point discrimination test (recorded in mm); - Pinprick test with VAS scoring (probe to apply stimulus was rated with VAS scale 0–5).	GaAlAs laser combined with LED (GRR) GaAlAs laser (Epic 10)	GRR laser: 904 and 650 nm, stable probe 10 mm in diameter, 50 mm penetration depth, 9 J intraoral, 16 J extraoral; 2. Epic10 940 nm, moving 15 mm diameter probe (intraoral), moving 35 mm × 8 mm 2.8 cm^2^ probe (extraoral), 5 J, 10 sessions over 5 weeks. Laser applied 5 min transmucosal, 5 min transcutaneous. One session lasted 10 min.	Brush test: - No significant difference between groups. 2. Two-point discrimination test: - LLLT showed significant difference compared to the vitamin group at the 4th assessment. 3. Pinprick test: A. Within group comparison (comparing T0-T5) - Significant improvement in all groups: points A, B, E, G, I, J - Significant improvement only in laser groups: points C, D - Significant improvement only in GRR laser group: point F. B. Between group comparison - Significant difference only at point “C” at 6th examination. All patients reported complete paresthesia resolution by 6 months post-BSSO complications.
Guarini [55]	Bilateral sagittal split osteotomy (BSSO)	The inferior alveolar nerve—the part it supplies	Five tests used: Visual Analog Scale (VAS); Sensitivity threshold test using nylon monofilament; Two-point discrimination using dry-point compass; Pain discrimination using dry-point compass; Thermal discrimination test for warm and cold stimuli.	GaAlAs diode laser (Flash Lase III; DMC Equipment, São Paulo, Brazil)	810 ± 20 nm; continuous wave, in contact, optical fiber probe with round tip of 0.6 cm diameter; 0.283 cm^2^ spot size, 0.353 W/cm^2^; 31.8 J/cm^2^; 270 s per session; 27 J total; 8 applications: days 1, 2, 3, 5, 10, 14, 21, 28 post-BSSO.	Two years post-BSSO: - sensitivity (VAS): 33.33% of laser group recovered sensitivity vs. 0% in control group - sensitivity threshold test: laser 69.7% vs. control 44.4% recovery - two-point discrimination: 69.70% recovered in laser group vs. 11.11% in control group - pain (VAS): 93.94% recovered in laser group vs. 55.56% in control one - thermal discrimination: cold: 100% recovery in both groups warm: 96.97% recovery in laser group vs. 66.67% in control group. Overall achieved recovery rate: 85% in laser group, 70–75% in control group. Recovery was similar in the first 28 days, after that period the laser group demonstrated superior regeneration.
De Oliveira [56]	- Orthognathic surgery; - 3rd molar extraction; - Dental implant placement; - Facial trauma; - Other minor surgeries (bone graft, residual root surgery, hyperplasia surgery, dental anesthesia)	The inferior alveolar nerve, superior alveolar nerve, infraorbital, lingual, maxillary nerve	Only subjective judgment of patients classified into 4 levels.	Primary laser: infrared. Secondary laser: red laser. (Photon Lase I and II DMC, São Carlos, SP, Brazil)	Standard protocol (primary laser): 808 nm; continuous wave contact mode, 100 mW; 100 J/cm^2^; 28 s/point, 1.0–1.5 cm point spacing; 2. Modified protocol (after 10 sessions if needed) 660 nm; other parameters unchanged.	Two factors correlate with the recovery rate: - Age (younger patients 14–25 had better outcomes); - Time interval between surgery and treatment (earlier intervention <30 days had better outcomes). Recovery outcomes: excellent 11.2%, good 39.2%, reasonable 31.2% and poor 18.4% of patients. Infrared laser was more effective than the red one. Once-weekly treatment showed better results than twice-weekly.
de Morais Filho [57]	Bilateral orthognathic surgery	Area of nose, nasal folds bilaterally, lower lip, chin.	Microbrush applicator to: - apply light touch to test areas of declared paresthesia; - marked boundaries of no sensation using toothpaste via Microbrush; - analyzed whether sensation would appear.	InGaAlP (Model XT, Sao Carlos, SP, Brazil)	808 ± 10 nm; continuous wave; 100 mW ± 20%, 35 J/cm^2^; 60 s per site at specific acupoints (ST5, ST6, CV24, GV26, LI4, LU7, ST36, ST45).	After 6 weeks of weekly LLLT the patient reported a sense of touch returning. Paresthesia showed reduction in marked area. Residual sensory deficits remained localized to chin and lower lip regions.
Miloro [58]	Bilateral sagittal split osteotomy (BSSO)	The inferior alveolar nerve and the part it supplies.	Three lever clinical neurosensory test: Level A: brush stroke directional discrimination and 2-point discrimination; Level B: contact detection; Level C: pin prick nociception and thermal discrimination. Subjective assessment: Visual analog scale (VAS).	Low- level GaAlAs diode laser	820–830 nm, 550 mW/cm^2^, 6 J for 90 s for each point.	Level A: Brush stroke directional discrimination: return of >90% of preoperative level by 14 days; 2-point discrimination: improvement after 14 days and return to normal after 2 months; Level B: Contact detection: improvement after 14 days and return to normal after 2 months; Level C: pin prick nociception and thermal discrimination: minimal neurosensory deficits- two cases of prolonged recovery by two months; VAS: rapid improvement; 50% deficit in 2 days and <15% in two months.
Santos [59]	Bilateral sagittal split osteotomy (BSSO)	Mandibular region	Semmes-Weinstein monofilament test	Diode laser	780 nm, 157.5 J/cm^2^, 90 s	Sensorineural recovery was noted in both sides with significant improvement on the experimental side (LLLT) during early postoperative sessions, particularly by the fifth session, though no regional differences were observed.
Mohajerani [60]	Sagittal split osteotomy (SSO)	Inferior alveolar nerve	Objective tests: Level A: brush stroke directional discrimination and 2-point discrimination; Level B: contact detection; Level C: pin prick nociception and thermal discrimination. Subjective test: Visual analog scale (VAS).	Low-level laser and LED	810 nm laser, 5 J/cm^2^, and 632 nm LED, 2 J/cm^2^	VAS scores improved in the laser group after 1 week, improvement in brush stroke and two-point discrimination tests was noted in 2 weeks; no differences in contact detection, pinprick, or thermal discrimination were observed at 6 months.
Khullar [61]	Sagittal split ramus osteotomy (SSO)	The inferior alveolar nerve (4 treatment points) and the part it supplies such as lip and chin area	Objective assessments: - Mechanoperception (Semmes Weinstein Monofilaments); - thermoception (Thermotester) to measure warm, cold, and thermal pain thresholds; - Standardized photographs to document the area of sensory damage Subjective assessments: - VAS for lip and chin area	GaAlAs (Photon plus GaAlAs diode laser, Rønvig A/S, Vejle, Denmark).	820 nm; continuous wave; 70 mW; approximately 0.13 cm^2^ spot size; 550 mW/cm^2^; 4 × 6 J per treatment, 20 sessions	Subjective results (VAS scale): - LLLT group showed significant improvement in lip and chin sensations; - The placebo group showed no significant improvement. Objective results: Mechanoreception: - LLLT group showed significant decrease in the area of sensory deficit, strong tendency toward improvement in the lip region; - The placebo group showed no significant improvement. 2. Thermoception results: neither group showed significant improvement in thermal sensitivity. All patients maintained a normal heat pain response throughout. However, it is noted that the most severely damaged areas did not show statistically significant improvement.
Eshghpour [62]	Bilateral sagittal split osteotomy (BSSO)	The inferior alveolar nerve and the part it supplies such as lip and chin area	Two-point discrimination test with two sharp needles on 6 points, applied before, after the surgical procedure and on 15, 30, 45, 60 days later	InGaAIP diode laser (Thor DD2 Control Unit, Thor, London, UK)	Intraoral: 660 nm, continuous wave, 200 mW, spot size at 1 cm approx 1.3 cm^2^, 2 J, 1.5 J/cm^2^, 4 points located 1 cm away from the surgical site, 10 s Extraoral: 660 nm, continuous wave, spot size approx 0.28 cm^2^, 200 mW, 2 J, 7 J/cm2, 8 points on ramus and body of mandible along the distribution of inferior alveolar nerve, 10 s, LLLT continuation: 660 nm, continuous wave, spot size approx 0.28 cm^2^, 200 mW, 2 J, 7 J/cm^2^, 10 s, 8 points on the path of inferior alveolar nerve parallel to the mandibular ridge, 4 on lower labial mucosa, 2 on lower lip, 9 on chin skin.	Before, immediately after surgery, and on 15 and 30 days after operation, there were no significant differences between placebo and the laser sides, but on days 45 and 60 there was better sensation of the chin and lower lip resulting in lower 2-point discrimination distance.

**Table 3:** Quality assessment of included studies.

Criteria	Study													
	de Oliveira [49]	Prazeres [50]	D’avilla [51]	Esmaeelinejad. [52]	Buysee Temrano [53]	Baydan [54]	Guarini [55]	de Oliveira [56]	de Morais Filho [57]	Miloro [58]	Santos [59]	Mohajerani [60]	Khullar [61]	Eshghpour [62]
**Minimum 10 subjects**	1	0	1	1	1	1	1	1	0	0	1	1	1	1
**Blinding**	1	0	1	1	1	1	1	0	0	0	1	1	1	1
**Sample size calculation**	1	0	1	1	0	1	0	1	0	0	1	1	0	1
**Randomization**	1	0	1	1	1	1	1	0	0	0	1	1	1	1
**Presence of a control group**	1	1	1	1	1	0	1	0	0	0	1	1	1	1
**Detailed description of PBM parameters (fluence, irradiance, dose per session, total dose)**	1	1	1	1	1	1	1	1	1	1	1	1	1	1
**Total**	6	2	6	6	5	5	5	3	1	1	6	6	5	6
**Risk of bias**	low	high	low	low	low	low	low	moderate	high	high	low	low	low	low

## 4. Discussion

Orthognathic surgery encompasses a range of procedures aimed at improving both functional outcomes and facial esthetics. Despite these therapeutic benefits, the procedures carry inherent risks, with neurosensory disturbances being among the most prevalent complications. To mitigate these issues and enhance postoperative recovery, various therapeutic approaches have been explored. Among these, low-level laser therapy (LLLT) has demonstrated promising efficacy in promoting neurosensory recovery. This systematic review evaluated fourteen studies investigating the application of LLLT in post-orthognathic surgery care. The included studies comprised eleven randomized controlled trials [49,50,51,52,53,54,55,59,60,61,62], one retrospective analysis [56], one case report [57], and one prospective observational study [58]. Bilateral sagittal split osteotomy (BSSO) was the predominant surgical procedure, featured in seven studies [53,54,55,57,58,59,62], while sagittal split osteotomy (SSO) was utilized in three. Clinical outcomes indicated recovery rates of up to 85% in laser-treated groups, compared to 70–75% in control groups [55]. Early intervention, particularly within 30 days post-surgery and weekly treatment sessions, were associated with greater efficacy [56]. Studies employing a split-mouth design [53,59,62] reported significantly faster recovery in laser-treated sites compared to untreated areas [53]. Follow-up durations ranged from immediate postoperative assessments to two years [55], with complete resolution of paresthesia observed at six months in one laser-treated cohort [54].

Paresthesia treatment is a common postoperative complication following maxillofacial and orthognathic surgeries, and various studies demonstrated significant success with its ability to promote nerve regeneration and resolving paresthesia [4,26,36,44]. Alternative therapeutic approaches include acupuncture [20], electroacupuncture [21], and pharmacological interventions. Notably, steroid administration has shown potential for neurosensory recovery [45,63,64], while combination therapy using uridine triphosphate, cytidine monophosphate, and hydroxocobalamin has also been proposed as a promising option [65]. It should be mentioned that the anatomical regions most commonly affected by paresthesia align with the distribution of the inferior alveolar nerve, as highlighted in several studies [49,52,53,54,55,56,58,60,61,62]. Other studies, however, examined broader facial areas, including both the upper and lower lips along with the chin [50], extensive regions spanning from the preauricular area to the mandibular body and paranasal regions [51], and areas such as the nose, nasal folds, lower lip, and chin [57]. The biological mechanism of photobiomodulation (PBM) underlying its efficacy involves the enhancement of cellular energy production through increased adenosine triphosphate (ATP) synthesis [21,26,27] and the mitigation of oxidative stress [29,30]. Additionally, PBM facilitates tissue repair by augmenting mitochondrial function [21,26] and stimulating nitric oxide production [32]. In the context of orthognathic surgery, its clinical benefits include reduced postoperative pain and swelling [33,39], accelerated wound healing [33,34,39], and improved neurosensory recovery [36,38]. The effectiveness of PBM is significantly influenced by the careful selection of laser parameters [41] and the timing of application, with immediate postoperative use producing the most favorable outcomes [37,43].

Pain is one of the most significant and distressing postoperative complications following orthognathic surgery and, indeed, most surgical procedures. It is such a consistent and inherent aspect of surgical intervention that it is almost not classified as a complication. Low-level laser/light therapy has shown promising results in mitigating acute postoperative pain, achieving reductions of up to 50% within the first two days after surgery while also accelerating recovery. Studies included in this review that assessed pain outcomes [51,53,55,57,58,60] consistently reported both short- and long-term improvements in Visual Analog Scale (VAS) scores, aligning with findings from other reviews on the subject [5]. Pain management often relies on pharmacological interventions, with opioid prescriptions being the most common approach. However, while opioids are effective, their use carries the significant risks of dependency, addiction, and abuse, posing a major public health challenge in the United States [66]. Alternative medications have shown varying degrees of success [67], but all pharmacological treatments are associated with potential side effects [68]. Among these alternatives, melatonin has demonstrated promise, showing similar reductions in oxidative stress to those observed with photobiomodulation [69]. Preemptive analgesia is another effective strategy for managing pain and reducing the need for painkillers; however, it remains more invasive compared to the significantly less invasive and highly effective LLLT [70]. Furthermore, patients undergoing LLLT report higher satisfaction [53], attributed to reduced pain perception, which correlates with improved physical health and quality of life following major surgeries.

Neurosensory disturbance following orthognathic surgery is primarily characterized by changes in, or a complete loss of, sensitivity in areas supplied by branches of the trigeminal nerve, most commonly the inferior alveolar nerve after bilateral sagittal split osteotomy (BSSO) [70]. Preoperative factors and surgical technique employed significantly influence postoperative neural outcomes, with piezosurgery demonstrating superior results [71]. Numerous treatment protocols have been proposed to enhance sensory recovery in affected areas, though complete recovery remains uncertain. Combined studies reviewed here indicate that low-level laser/light therapy (LLLT) consistently accelerates improvements in sensory recovery, including heat/cold perception, pain stimulus response, and reductions in neurosensory disturbances, compared to control groups. Notably, the study by F. de Oliveira et al. [49] was an exception, showing no significant differences. However, in cases of severe nerve damage, no significant improvement was observed [57,61]. These findings align with previous reviews of LLLT in neurosensory disturbances, such as the one conducted by Firoozi P. et al. [72]. A study by Navarro-Fernández G. et al. [63], which compared various therapeutic modalities, found moderate evidence supporting the efficacy of laser and LED therapy in sensitivity rehabilitation, while halotherapy showed no benefit [64]. The most recent study by Pourdanesh F. et al. [73] highlighted the effectiveness of transcutaneous electrical nerve stimulation, corroborating the findings of F. de Oliveira et al. [45]. Pharmacological interventions following surgery show mixed results. Positive outcomes have been reported with melatonin, which aids sensitivity recovery [69]. Steroids demonstrated neutral effects on sensitivity but contributed to edema reduction [65,74,75]. Conversely, combination therapy using uridine triphosphate (UTP), cytidine monophosphate (CMP), and hydroxocobalamin (vitamin B12) showed no significant improvement in neurosensory recovery [76]. More direct interventions, such as stellate ganglion blockade [77], have demonstrated effectiveness. However, less invasive approaches like Xenon light irradiation and other photobiomodulation therapies reviewed here are advantageous, being easier to perform, better tolerated by patients, and associated with fewer risks.

Although many studies were included in this review, certain limitations were identified. The scope was restricted to articles published in English, and not all available databases were comprehensively searched, potentially leading to selection bias. Additionally, only 50% of the included studies were classified as high-quality, while nearly 30% were considered low-quality. There is a clear need for further clinical trials with more standardized and precise treatment protocols. These should account for consistent laser parameters—such as wavelength, dosage, and power—and uniform time spans for irradiation across the pre-, intra-, and postoperative periods. Future studies should also adopt standardized assessments for neurosensory disorder testing, enabling consistent comparisons and facilitating advancements in the field. While some studies included overlapping assessments, none encompassed all the relevant tests comprehensively. Moreover, the diversity in the types of surgeries performed and the anatomical regions targeted adds complexity to the analysis. The inferior alveolar nerve was the most frequently addressed nerve in the studies, but the application sites varied significantly. Considering these limitations, further research is essential to develop a precise and standardized laser/light therapy protocol for treating neurosensory disturbances in orthognathic surgery patients. Such efforts are crucial for maximizing the therapeutic potential of this promising modality. Moreover, the considerable heterogeneity among the included studies precludes the possibility of conducting a meta-analysis. To obtain more precise results and facilitate a meta-analysis, additional studies with greater homogeneity in terms of laser types and their parameters are needed.

## 5. Conclusions

Based on the reviewed literature, low-level laser therapy (LLLT) is a safe and effective modality for enhancing neurosensory recovery following orthognathic surgery, particularly when initiated early and administered consistently. While outcomes may vary depending on individual factors and specific treatment protocols, 13 out of 14 reviewed studies indicate that LLLT promotes the restoration of sensory function, especially when infrared wavelengths are employed with regular treatment sessions. However, the lack of standardized protocols and the limited number of large, controlled trials highlight the need for further research to establish an optimal treatment regimen.

## Figures and Tables

**Table 1:** General characteristics of studies.

Study	Aim of the Study	Material and Methods	Results	Conclusions
Oliveira [49]	To investigate the possibility of promotion of tactile and pain sensitivity return in patients submitted to orthognathic surgery by electroacupuncture and laseracupuncture.	A 30-patient randomized blinded trial comparing electrostimulation (Group 1) and 780 nm diode laser on acupuncture points (Group 2) after orthognathic surgery with genioplasty. Half-face treatment, twice a week. Measured tactile sensitivity (mechanical brush + 2-point discrimination) and pain (pulp electrical test) pre-surgery and 4 months post-op.	No statistically significant differences among the groups for the tests except tactile test using brush on the lower lip and chin between group 1 and others.	Laser-acupuncture does not accelerate the return of sensitivity after orthognathic surgery and genioplasty, but electrostimulation does.
Travassos Prazeres [50]	Treatment and preventions of paresthesias using 830 nm infrared laser on patients submitted to orthognathic surgery.	The 2 patients control and 4 patients experimental group after orthognathic surgery received 830 nm diode laser applications starting transoperative and for 12 sessions twice a week. Superficial, deep, and thermal sensitivity was tested, and a paresthesia evaluation was performed.	All patients had paresthesia postoperatively, but the experimental group showed faster reduction in paresthesia than the control group.	Laser therapy could be an effective way of paresthesia treatment in patients undergoing orthognathic surgery.
D’avilla [51]	Effectiveness of 940 nm laser therapy in recovery of patients submitted to orthognathic surgery.	Double-blinded randomized clinical trial, 10 control and 10 laser patients after orthognathic surgery, irradiated with 940 nm diode laser after surgery, 24 h, 48 h, twice a week until 30 days (11 sessions total); collected pain data with VAS, edema data, trismus data, paresthesia weekly with the brush touching skin of lower jaw.	Laser group reported lower pain from 24 h up to week 3, higher mouth opening after 2 and 4 weeks, higher positive responses till week 4 in the sensitivity recovery, but without significant difference between the groups during the experimental periods.	Photobiomodulation therapy using a 940 nm laser could reduce trismus and pain after orthognathic surgery.
Esmaeelinejad [52]	Possibility of improved recovery from neurosensory disturbances with the low-level laser therapy in patients undergoing sagittal split ramus osteotomy.	Double-blinded randomized clinical study 20 control and 20 test group irradiated with 810 nm diode laser, on days 0, 1, 2, 3, and every other day for the next 2 weeks (total 10 sessions); mechanoreceptor sensory and thermal, satisfaction of the patient tests were conducted; mapping of the affected skin area was created immediately, 3, 6, and 12 months after the surgery.	Laser group after one year showed significantly better distinction of two separate sharp points, positive contact direction test, higher satisfaction, and the whole test group was able to identify a touch with a sharp needle, unlike the control group. After one year both patients’ groups were able to detect the cool and heat.	LLLT could improve recovery from neurosensory disturbance in patients submitted to orthognathic surgeries, like split ramus osteotomy.
Buysee Temrano [53]	Effect of LLLT on neurosensory recovery of patients undergoing sagittal osteotomy of the mandible.	The 12 patients after orthognathic surgery were blinded to the choice of one half of the face as control and the other half treated with low-intensity infrared GaAlAs 808 nm lasers, following the course of the inferior dental nerve, 2–3 sessions per week starting 48 h after surgery for minimum 10 sessions. Pain (VAS), mechanical (touching and brushing) and thermal (gutta-percha, Endo-frost) test were conducted in the 1st, 4th, 7th, and 10th session.	Treated side with laser presented faster recovery, better sensibility recovery for all stimuli, higher perception of pain and thermal stimulus. Lips and teeth had better recovery index than the chin.	The 808 nm LLLT could accelerate recovery of post operative neurosensory disturbances in patients being submitted to orthognathic surgeries
Baydan [54]	To evaluate treatment outcomes of two different laser protocols versus vitamin B complex for post-BSSO lip paresthesia.	Examined 30 patients after BSSO with lip paresthesia randomized into: GRR laser (904/650 nm, n = 10), Epic10 laser (940 nm, n = 10), and Vitamin B (n = 10) groups. Laser groups received 10 sessions while the vitamin group had 30-day supplementation. Assessment included two-point discrimination, brush test and pinprick testing with VAS scoring. There were 9 lip-chin regions assessed at six time points.	Laser groups showed better recovery rates than vitamin B group. Points A, B, E, G, I, J improved across all groups. GRR laser showed best recovery at point C. Points C, D improved in laser groups only, while point F in GRR group only. LLLT outperformed vitamin B group at 4th assessment. All patients reported complete paresthesia resolution by 6 months post-BSSO complications.	Both laser therapies and vitamin B showed positive effects on nerve regeneration, but laser treatments demonstrated superior outcomes.
Guarini [55]	The research investigated the long-term outcomes of photobiomodulation treatment in post-BSSO patients presenting with inferior alveolar nerve dysfunction, using a 24-month monitoring period.	A 2-year follow-up study comparing photobiomodulation therapy (n = 33) vs. placebo (n = 9) for post-BSSO neurosensory deficit. GaAlAs diode laser applied at 3 anatomical sites bilaterally: mandibular foramen, mental foramen, and buccal osteotomy region. The 8 applications were performed (days 1, 2, 3, 5, 10, 14, 21, and 28 postoperatively). The 5 neurosensory tests were performed: VAS for pain and sensitivity, sensitivity threshold test, two-point discrimination, pain discrimination, and thermal discrimination. Tests were conducted from 24 h pre-op to 2 years post-op.	The laser group showed better neurosensory recovery compared to controls. Normal sensitivity was achieved in 40.74% of laser-treated patients (vs 0% control), with 69.7% recovering two-point discrimination and 93.94% reporting normal pain response. Thermal discrimination showed improvement but without statistical significance.	GaAlAs laser photobiomodulation proved more effective than placebo for neurosensory rehabilitation. Treated patients exhibited 85% recovery of nerve function, significantly exceeding the control group’s 70–75% restoration rate.
de Oliveira [56]	To assess laser therapy’s role in accelerating and recovering neurosensory following orthognathic and minor oral surgical interventions.	Retrospective study analyzed 125 clinical records. Patients divided into groups based on age, period between surgery and laser therapy, treatment frequency, treatment outcomes and guided by protocol: maintaining the standard protocol with 808 nm laser vs. modified protocol after 10 sessions using 660 nm laser (other parameters unchanged).	The bidimensional analysis revealed highest recovery rates in younger patients (14–25 years), males, and cases treated within 30 days post-surgery. Orthognathic and trauma-related cases showed better outcomes than implant-associated paresthesia. Weekly treatment had greater efficacy than bi-weekly ones, and the standard 808 nm protocol showed better results than the modified one.	LLLT using 808 nm laser is effective in treating post-surgical paresthesia. Recovery outcomes correlated with patient age and early intervention timing.
de Morais Filho [57]	Effectiveness of laser acupuncture (LA) in treating facial paresthesia in a 28-year-old female patient after orthognathic surgery. The patient experienced loss of touch sensitivity in multiple areas including nose, nasal folds (on both sides), lower lip, and chin region.	Sensory assessment was performed using Microbrush applicators, mapping affected areas. The boundaries of the patient’s administered sensory loss were marked with a toothpaste via Microbrush. Treatment consisted of weekly InGaAlP 808 nm laser applications specific acupoints (ST5, ST6, CV24, GV26, LI4, LU7, ST36, ST45).	The 6 weeks of weekly laser acupuncture resulted in improvement of sensory function, with remaining residual paresthesia in mental and labial regions.	This case report demonstrates the success of laser acupuncture in treating paresthesia after orthognathic surgery. However, further controlled studies are needed to verify its effectiveness.
Miloro [58]	To assess the effect of pre- and postoperative LLLT on neurosensory recovery after bilateral sagittal split osteotomy (BSSO).	The 6 patients subjected to BSSO surgery were enrolled in a preoperative neurosensory test. After the surgery, the LLLT was applied using 820–830 nm GaAlAs. The laser treatment was performed intraorally and extraorally in seven sessions (immediately after the surgery, 6 h, 24 h, 2, 3, 4, and 7 days after). On days 14 and 28 the neurosensory evaluation was conducted.	LLLT significantly accelerated neurosensory recovery after surgery. Sensitivity to brush strokes approached normal within 14 days, while 2-point and contact detection improved by 14 days and returned to near-normal by 2 months. Minimal deficits in temperature and pain response lasted up to 2 months in some cases.	LLLT can significantly improve the speed and extent of neurosensory recovery after BSSO.
Santos [59]	To investigate the effect of LLLT on sensorineural deficiency recovery after bilateral sagittal split osteotomy (BSSO).	A group of 20 patients underwent the BSSO surgery and received 780 nm diode laser LLLT on one side of the mandible and placebo on the other side. Patients were divided into two groups- group 1 short postoperative period (30 days) and group 2 -patients experiencing lasting sensory issues in the later postoperative period (6 months to 1 year). Each patient receives five sessions with 3–4 weeks intervals. The laser irradiation was applied extra and intraorally.	Both the control and experimental sides showed postoperative improvement, with the laser-treated side demonstrating a significant enhancement in sensorineural recovery across sessions in both patient groups.	The use of LLL is effective in treating sensory disorders after BSSO surgeries, especially in the short postoperative period.
Mohajerani [60]	To examine the combined effect of LLLT and LED on inferior alveolar nerve disorders recovery following mandibular sagittal split osteotomy (SSO).	A group of 20 patients was divided in two groups (experimental and control) were subjected to the study after SSO surgery. The experimental group received a combined application of 810 nm LLL and 632 nm LED beam. The device was applied intra and extraorally. Each point received laser application on the 1, 2, 3, 7, 14, and 28 days after surgery. Patients were evaluated by VAS, brush stroke, 2-point discrimination, contact detect detection, pinprick nociception, and thermal discrimination tests.	As the neurosensory recovery was assessed by six tests, in all tests all laser groups showed a significant improvement after 1 and 2 weeks and 6 months.	A combination of LLLT and LED can improve neurosensory recovery after orthognathic SSO surgery.
Khullar [61]	To evaluate both the objective and subjective outcomes of LLLT in patients with paresthesia that underwent SSO. The study assessed whether objectively verified improvements in sensory function correlated with patients’ subjective perception of improvement after treatment.	A double-blinded trial was conducted on 13 patients (20–55 years) with 2-year post-SSO neurosensory deficits. Patients were randomly divided into the LLLT group (n = 8, GaAlAs 820 nm, 20 sessions at 4 standardized points along the inferior alveolar nerve) and placebo group (n = 5). Assessments included objective mechanoperception, thermoception and subjective measures using VAS.	The LLLT group showed significant improvement in subjective (lip, chin sensation) and objective nerve function. Superior lip sensitivity improvement was confirmed by both objective and subjective tests. Thermal sensitivity remained unchanged in both groups. All patients maintained normal protective heat pain responses. The placebo group showed no significant improvements in any parameters.	While improvement trends were observed in mechanical sensitivity, particularly in the lip region, the most severely damaged areas did not show statistically significant improvement perception. Nevertheless, patients reported significant subjective improvements, and objective measurements showed reduced areas of sensory deficit.
Eshghpour [62]	To evaluate the effectiveness of low level laser therapy (LLLT) in treating sensorineural deficiency in patients undergoing bilateral split sagittal osteotomy (BSSO).	Double-blind, randomized, split-mouth trial on 16 patients, LLLT with intraoral 660 nm InGaAIP diode laser and extraoral 810 nm InGaAIP diode laser at 24, 48, 72 h after operation and extraoral continuation for 3 weeks twice a week along inferior alveolar nerve path. Assessments included objective mechanoreception tested with 2-point discrimination test up to 60 days after operation.	On the side treated with LLLT there was significantly lower 2-point discrimination distance on days 45 and 60 after operation, but there was no significant difference between placebo and laser side before, after surgery, and on 15 and 30 days later.	LLLT performed could be an effective treatment for neurosensory disturbances following BSSO and could accelerate the recovery.

## Data Availability

Data supporting the findings of this study are available within the article.
